# Associations of Filaggrin Gene Loss-of-Function Variants with Urinary Phthalate Metabolites and Testicular Function in Young Danish Men

**DOI:** 10.1289/ehp.1306720

**Published:** 2014-01-03

**Authors:** Ulla Nordström Joensen, Niels Jørgensen, Michael Meldgaard, Hanne Frederiksen, Anna-Maria Andersson, Torkil Menné, Jeanne Duus Johansen, Berit Christina Carlsen, Steen Stender, Pal Bela Szecsi, Niels Erik Skakkebæk, Ewa Rajpert De Meyts, Jacob P. Thyssen

**Affiliations:** 1 Department of Growth and Reproduction, Copenhagen University Hospital (Rigshospitalet), Copenhagen, Denmark; 2 Department of Clinical Biochemistry, and; 3 National Allergy Research Centre, Department of Dermato-Allergology, Copenhagen University Hospital Gentofte, Copenhagen, Denmark

## Abstract

Background: Filaggrin is an epidermal protein that is crucial for skin barrier function. Up to 10% of Europeans and 5% of Asians carry at least one null allele in the filaggrin gene (*FLG*). Reduced expression of filaggrin in carriers of the null allele is associated with facilitated transfer of allergens across the epidermis. We hypothesized that these individuals may have increased transdermal uptake of endocrine disruptors, including phthalates.

Objectives: We investigated urinary excretion of phthalate metabolites and testicular function in young men with and without *FLG* loss-of-function variants in a cross-sectional study of 861 young men from the general Danish population.

Methods: All men were genotyped for *FLG* R501X, 2282del4, and R2447X loss-of-function variants. We measured urinary concentrations of 14 phthalate metabolites and serum levels of reproductive hormones. We also evaluated semen quality.

Results: Sixty-five men (7.5%) carried at least one *FLG*-null allele. *FLG*-null carriers had significantly higher urinary concentrations of several phthalate metabolites, including a 33% higher concentration of MnBP (mono-***n***-butyl phthalate; 95% CI: 16, 51%). *FLG*-null variants were not significantly associated with reproductive hormones or semen quality parameters.

Conclusion: This study provides evidence that carriers of *FLG* loss-of-function alleles may have higher internal exposure to phthalates, possibly due to increased transepidermal absorption. *FLG* loss-of-function variants may indicate susceptible populations for which special attention to transepidermal absorption of chemicals and medication may be warranted.

Citation: Joensen UN, Jørgensen N, Meldgaard M, Frederiksen H, Andersson AM, Menné T, Johansen JD, Carlsen BC, Stender S, Szecsi PB, Skakkebæk NE, Rajpert-De Meyts E, Thyssen JP. 2014. Associations of filaggrin gene loss-of-function variants with urinary phthalate metabolites and testicular function in young Danish men. Environ Health Perspect 122:345–350; http://dx.doi.org/10.1289/ehp.1306720

## Introduction

In human stratum corneum of the skin, protein-enriched corneocytes are embedded in a lipid-rich matrix that impedes egress of water and penetration of pathogenic microorganisms, allergens, and noxious chemicals. To maintain skin hydration, abundant intracellular filaggrin proteins are hydrolyzed into amino acids and their deiminated products, collectively referred to as the “natural moisturizing factors” (Gruber et al. 2011). Filaggrin deficiency due to the presence of one or more loss-of-function variants in the filaggrin gene (*FLG*) is observed in approximately 10% of lightly pigmented Europeans and in a slightly lower proportion of Asians (Irvine et al. 2011). These variants cause ichthyosis vulgaris (Smith et al. 2006), which is characterized by xerosis, scaling, and keratosis pilaris as well as palmar and plantar hyperlinearity. Permeation of allergens seems to be increased in filaggrin-depleted skin (Fallon et al. 2009; Gruber et al. 2011; Scharschmidt et al. 2009), and an increased risk of atopic dermatitis, asthma, rhinitis, food allergies, and nickel sensitization have been observed (Brown et al. 2011; Palmer et al. 2006; Thyssen et al. 2010). Clinical trials are currently being conducted to study whether primary prevention of atopic disorders is possible using topical therapy with moisturizers (Irvine et al. 2011).

Diesters of 1,2-benzenedicarboxylic acid, commonly referred to as phthalates, are man-made chemicals used in a wide range of consumer products, including moisturizers and other cosmetics. Although phthalates are rapidly metabolized and excreted in the urine following absorption, humans are continuously exposed by skin contact with, for example, cosmetics, fragrances, solvents, and plastics (Wittassek et al. 2011), as well as through the diet and via inhalation. An inverse association between phthalate exposure and markers of testicular function has been reported in some human studies (Meeker et al. 2009; Mendiola et al. 2011). Animal studies have established that certain phthalates act as endocrine disruptors, resulting in developmental abnormalities of the male reproductive tract as well as inhibition of testicular testosterone production in prenatally or perinatally exposed animals (Boberg et al. 2011; Gray et al. 2000). Although the pathogenic effects of phthalate exposure on testicular function in humans is not yet clear, certain phthalates inhibit testosterone synthesis in cultured adult human testes at concentrations estimated to be within the range observed in epidemiological studies (Desdoits-Lethimonier et al. 2012).

In the present study, we addressed the question of whether male *FLG*-null carriers would have increased internal exposure to phthalate metabolites, and if so, whether this would affect testicular function.

## Methods

*Study population*. All Danish men are called to a compulsory examination the year of their 18th birthday to determine fitness for military service. On the day they went for this examination, men residing in the Copenhagen, Denmark, area were asked to participate in a study on semen quality. A total of 881 men volunteered during 2007–2009 for a study on urinary phthalate excretion and testicular function (Joensen et al. 2012). The participation rate was approximately 30%, which is higher than in other population-based semen-quality studies (Jørgensen et al. 2002). Basic study details have been described previously (Jørgensen et al. 2012). Each man underwent a physical examination focusing on reproductive development, completed a questionnaire, and gave samples of spot urine, semen, and blood, in most cases all within 1 hr. Ejaculation abstinence period and time of blood sampling were recorded. All urine, semen, and blood samples were collected between 0840 hours and 1230 hours (median time, 1000 hours). In 2011, *FLG* genotyping was performed according to a protocol developed for the present study. DNA samples from 13 of the 881 men were missing or of poor quality; for another 7 men, the urine sample was too small to analyze osmolality. This left 861 men with complete data on *FLG* variant status and other main outcomes. All laboratory analyses were carried out on coded samples.

The research protocol was approved by the Danish National Committee on Biomedical Research Ethics (no. H-KF-289428). Participants gave written informed consent before participation.

*Questionnaire*. Questions included information on lifestyle factors (e.g., smoking and alcohol consumption) and medical history. The responses were reviewed with the participants to clarify missing or equivocal information. Participants provided information on use of prescription and nonprescription medication within the past 3 months, but were not asked to further specify the time of use. Thus, men reporting medicine intake within the past 3 months may not have taken any within ≥ 24 hr. Ethnicity was deduced from the self-reported country of birth of the participant and his parents; 83 men were categorized as other than European [i.e., they themselves or one or both of their parents were born outside of European countries (20 from African countries, 16 from Asian countries, 4 from Greenland, 11 from Latin American countries, and 32 from Middle Eastern countries)]. Participants were asked to report any previous diseases, with the focus on reproductive disorders. The questionnaire included a specific question about previous diagnosis of asthma and hay fever but no specific questions about eczema, food allergies, or use of moisturizers or other personal care products.

FLG *genotyping*. DNA was purified from blood samples and stored at –80°C until analysis. *FLG* genotyping was performed according to the method of Meldgaard et al. (2012), with slight modifications. Briefly, three regions covering the mutations R501X, 2282del4, and R2447X of the *FLG* were asymmetrically amplified from genomic DNA by polymerase chain reaction (PCR) using DNA-tagged allele-specific primers. The obtained single-stranded PCR products were hybridized with MagPlex-C micro beads (Luminex, Austin, TX, USA) carrying the DNA tags as probes, and subsequently analyzed on a Bio-Plex 200 system (Bio-Rad, Hercules, CA, USA). Deviation from the Hardy-Weinberg equilibrium was tested for all three *FLG*-null variants using a free online calculator (Online Encyclopedia for Genetic Epidemiology 2008). Participants were classified as *FLG*-null carriers if they carried at least one of the null *FLG* alleles that were evaluated (R501X, 2282del4, or R2447X).

*Osmolality analyses*. To assess urinary dilution, we measured urinary osmolality using the freezing point depression method and an automatic cryoscopic osmometer (Osmomat® 030; Gonotec, Berlin, Germany). For every 12 samples measured, a standard urine pool was also measured. Mean urinary osmolality for this standard pool (*n* = 73) was 0.83 Osm/kg with a relative standard deviation (RSD) of 1.63%. Concentrations of phthalate metabolites were adjusted for osmolality by dividing the measured metabolite concentration with the urinary osmolality of that sample and multiplying the result by the median osmolality of the population (0.86 Osm/kg). Because urine density is close to 1 kg/L, 1 Osm/kg was assumed to equal 1 Osm/L. Only samples with phthalate metabolite concentrations > LOD (limit of detection) were adjusted in this way; samples < LOD were left unadjusted for osmolality.

*Urinary phthalate metabolite analyses*. Urine samples were collected in polyethylene cups. From each sample, 15 mL was decanted into a 20-mL glass scintillation vial and the top of the vial packed with aluminum foil; the vials were stored at –20°C until analysis. Samples were analyzed for content of 14 phthalate metabolites (Table 1) by liquid chromatography–tandem mass spectrometry (LC-MS/MS) preceded by enzymatic deconjugation and solid phase extraction. Methods for sample preparation, standard solutions, quality controls, instrumental analysis, and general method validation have previously been described (Frederiksen et al. 2010). Urine samples were analyzed in 25 batches over 11 weeks. Each batch included standards for calibration curves and approximately 35 coded samples plus 2 blanks, 2 urine pool controls, and 2 urine pool controls spiked with low levels of phthalate standards. The interday assay variation, expressed as the RSD, was < 20% for all analytes except MCPP (mono-3-carboxypropyl phthalate; 22%) and MiNP (mono-isononyl phthalate; 26%). Recovery of spiked control samples was > 90% for all analytes except MnBP (mono-*n*-butyl phthalate; 88%) and MCPP (85%). LODs were established as previously described (Frederiksen et al. 2010). Results below LOD were assigned a value of the LOD divided by the square root of 2.

**Table 1:** “Parent” phthalate diesters to which humans are exposed, and their metabolites that are measurable in urine.

Phthalate group	Parent phthalate diesters		Measurable urinary metabolites		LOD (ng/mL)
LMW	DEP	Diethyl phthalate	MEP	Monoethyl phthalate	0.46
	DnBP	Di-*n*-butyl phthalate	MnBP	Mono-*n*-butyl phthalate	0.57
	DiBP	Diisobutyl phthalate	MiBP	Monoisobutyl phthalate	0.55
	BBzP	Butylbenzyl phthalate	MBzP	Monobenzyl phthalate	0.25
HMW	DEHP	Di(2-ethylhexyl) phthalate	MEHP	Mono(2-ethylhexyl) phthalate	0.63
			MEHHP	Mono(2-ethyl-5-hydroxyhexyl) phthalate	0.30
			MEOHP	Mono(2-ethyl-5-oxohexyl) phthalate	0.30
			MECPP	Mono(2-ethyl-5-carboxypentyl) phthalate	0.17
	DOP	Dioctyl phthalate	MOP	Mono-*n*-octyl phthalate	0.15
			MCPP^*a*^	Mono-3-carboxypropyl phthalate	0.14
	DiNP	Diisononyl phthalate	MiNP	Mono-isononyl phthalate	0.15
			MHiNP	Mono(hydroxy-isononyl) phthalate	0.12
			MOiNP	Mono(oxo-isononyl) phthalate	0.11
			MCiOP	Mono(carboxy-isononyl) phthalate	0.05
Abbreviations: LMW, low-molecular-weight phthalates (often found in personal care products); HMW, high-molecular-weight phthalates (used as plasticizers; infrequently found in personal care products). ^***a***^MCPP is a metabolite of DOP, and is also a minor metabolite of diisooctyl phthalate, diisodecyl phthalate, DiNP, DEHP, BBzP and DBP (dibutyl phthalate).					

Concentrations of phthalate diesters were calculated by multiplying the measured molar urinary concentration by the molar weights of the respective diesters. The sum of the two dibutyl phthalate (DBP) isomers [di-*n*-butyl phthalate (DnBP) and diisobutyl phthalate (DiBP); ΣDBP_(i+n)_] and the sums of DEHP [di(2-ethylhexyl) phthalate] and DiNP (diisononyl phthalate) metabolites (ΣDEHPm and ΣDiNPm) were calculated by adding the molar concentration of metabolites and multiplying the sum by the molar weights of the respective diesters (Frederiksen et al. 2010). For DEHP and DiNP, the ratio between the molar concentration of the primary metabolite {MEHP [mono(2-ethylhexyl) phthalate] and MiNP, respectively} and the total molar concentration of all four metabolites was calculated as the percentage of MEHP (%MEHP) and MiNP (%MiNP) as a measure of an individual’s pattern of metabolism of DEHP and DiNP (Joensen et al. 2012). In the present study, we distinguish between low-molecular-weight (LMW) and high-molecular-weight (HMW) phthalate diesters and their metabolites, where DEP (diethyl phthalate), DiBP, DnBP, and BBzP (butylbenzyl phthalate) are LMW phthalates and DEHP, DOP, and DiNP are HMW phthalates.

*Reproductive hormone analyses*. Serum was stored at –20°C until analysis, and was subsequently analyzed in batches. We measured concentrations of follicle-stimulating hormone (FSH), luteinizing hormone (LH), and sex hormone-binding globulin (SHBG) using a time-resolved fluoroimmunoassay (Delfia; Wallac, Turku, Finland). Total testosterone and estradiol were determined by radioimmunoassay (Coat-a-Count, Diagnostic Products Corporation, Los Angeles, CA, USA; and Pantex, Santa Monica, CA, USA). Inhibin-B was determined using a double antibody enzyme-immunometric assay (Oxford Bioinnovation or DSL Beckman, USA). Between 2008 and 2010, slight adjustments were made to the inhibin-B analysis, and the LOD was lowered from 20 pg/mL to 7 pg/mL. The intra- and interassay coefficients of variation (CVs) for measurement of FSH, LH, and SHBG were < 6%, and those for total testosterone were < 10%. Intra- and interassay CVs for estradiol were 8% and 13%, respectively, and for inhibin-B were 15% and 18%. We calculated free testosterone (cFT) from total testosterone and SHBG, assuming a fixed albumin level of 43.8 g/L, as described by Vermeulen et al. (1999). Hormone ratios were calculated by simple division.

*Semen analysis*. Semen volume was assessed by weight, and sperm concentration was determined using a Bürker-Türk hemocytometer. We determined the total sperm count (semen volume × sperm concentration) and the percentage of progressively motile spermatozoa. Morphology slides were fixed, Papanicolaou-stained, and assessed according to strict criteria as described by Menkveld et al. (1990). Semen analysis was performed in accordance with World Health Organization guidelines (World Health Organization 2010) and has been described in detail previously (Jørgensen et al. 2002).

*Statistics*. Crude differences between the outcomes (phthalate metabolites, hormones, and semen-quality variables) for *FLG*-null and wild-type (WT) carriers were first analyzed using the Mann-Whitney test for nonnormally distributed continuous variables. We estimated associations between *FLG* genotype and outcomes using multivariate linear regression models. Dependent variables were either natural logarithm (ln) transformed (all phthalate metabolites, hormones, hormone ratios, and semen volume), cubic root transformed (sperm concentration, total sperm count, total normal spermatozoa), squared (progressively motile spermatozoa), or square-root transformed (morphologically normal spermatozoa) to achieve normality of distribution of the residuals. Adjusted means and 95% confidence intervals (CIs) were calculated by back-transformation of the estimates from the linear regression analyses.

Potential confounders were identified and included as model covariates if they were significant predictors of the outcome (*p* < 0.05) in a bivariate linear regression model. For ease of interpretation, if a covariate was a significant predictor of outcome in several models with hormone levels as an outcome, that predictor was included for all models with hormones as an outcome. Likewise, a significant predictor of concentration of several phthalate metabolites would be included in all models with phthalate metabolite levels as an outcome. In models of associations between hormone levels and *FLG* genotype, the following confounders were included as continuous variables: body mass index (BMI), smoking (number of cigarettes per day), alcohol consumption (units consumed in the week before participation), time of blood sampling, and age. The type of assay was also included as a dichotomous variable in the inhibin-B models. In models of associations between *FLG* genotype and semen variables, we adjusted for ejaculation abstinence time (for models of semen volume, sperm concentration, and total sperm count, which were positively associated with abstinence time) or time from ejaculation to semen analysis (for models of the percentage of progressively motile spermatozoa, which was negatively associated with time from ejaculation to analysis). Percentage of morphologically normal spermatozoa was left unadjusted because none of the tested confounders were significant predictors of the outcome (*p* > 0.05). Models of phthalate metabolite levels and *FLG* genotype were adjusted for age, number of cigarettes per day, and year of participation. Participants evaluated in 2007 were more likely to carry null *FLG* alleles than participants tested in 2008 and 2009 (see Supplemental Material, Table S1), and mean urinary levels of some phthalate metabolites were also highest in 2007 (Supplemental Material, Figure S1). Year of participation was a significant predictor for several phthalate metabolites and was therefore included as a potential confounder in all models of associations between *FLG* genotype and urinary phthalate metabolites.

No data were missing for *FLG* genotype or for reproductive hormones. Data for other variables of interest are presented in Table 2, including the number of men with missing data for each variable; cases with missing data were excluded on an analysis-by-analysis basis.

**Table 2:** Basic characteristics, unadjusted serum concentrations of reproductive hormones, and semen quality parameters by *FLG* genotype in 861 men.

Variable	Missing (*n*)	Unadjusted median (5th, 95th)		Regression coefficient (95% CI)	*p-*Value^*a*^
		*FLG*-null	*FLG* WT		
Age (years)	4	19 (19, 21)	19 (18, 22)	NA	0.1^*b*^
BMI (kg/m^2^)	7	21.9 (19, 28)	22.6 (19, 29)	NA	0.04^*b*^
Alcohol use (units/week)	34	14 (0, 49)	12 (0, 42)	NA	0.9^*b*^
Smoking (no. of cigarettes/day)	13	0 (0, 15)	0 (0, 20)	NA	0.5^*b*^
Urinary osmolality (Osm/kg)	0	0.9 (0.4, 1.1)	0.9 (0.3, 1.1)	NA	0.07^*b*^
Testosterone (nmol/L)	0	19 (12, 31)	19 (11, 30)	–0.01 (–0.08, 0.07)^*c*^	0.9^*d*^
Estradiol (nmol/L)	0	75 (48, 127)	77 (45, 126)	0.01 (–0.07, 0.09)^*c*^	0.7^*d*^
SHBG (nmol/L)	0	29 (18, 50)	27 (12, 51)	–0.09 (–0.19, 0.01)^*c*^	0.07^*d*^
LH (IU/L)	0	3.1 (1.4, 6.4)	3.0 (1.4, 6.2)	–0.07 (–0.18, 0.04)^*c*^	0.2^*d*^
Inhibin-B (pg/mL)	0	188 (81, 298)	175 (98, 304)	–0.03 (–0.13, 0.07)^*c*^	0.6^*d*^
FSH (IU/L)	0	2.7 (1.2, 5.8)	2.3 (0.9, 5.6)	–0.14 (–0.28, 0.01)^*c*^	0.07^*d*^
cFT (ng/dL)	0	12 (7.9, 20)	12 (7.6, 21)	0.02 (–0.07, 0.10)^*c*^	0.7^*d*^
cFT/LH ratio	0	3.7 (1.9, 7.8)	4.1 (2.0, 8.6)	0.08 (–0.03, 0.20)^*c*^	0.2^*d*^
Testosterone/estradiol ratio	0	262 (150, 389)	242 (149, 382)	–0.02 (–0.09, 0.05)^*c*^	0.6^*d*^
Testosterone/LH ratio	0	5.5 (2.9, 12)	6.2 (3.0, 12)	0.06 (–0.04, 0.17)^*c*^	0.2^*d*^
Inhibin-B/FSH ratio	0	70 (12, 197)	79 (19, 303)	0.11 (–0.11, 0.33)^*c*^	0.3^*d*^
Semen volume (mL)	6	3.1 (1.3, 5.8)	3.2 (1.3, 6.3)	1.29 (1.16, 1.42)^*c*^	0.8^*e*^
Sperm concentration (10^6^/mL)	3	48 (1.9, 172)	48 (4.4, 167)	3.95 (3.65, 4.26)^*f*^	0.8^*e*^
Total sperm count (10^6^)	7	164 (5.6, 447)	142 (13, 537)	6.11 (5.66, 6.55)^*f*^	0.6^*e*^
Total normal sperm (10^6^)	19	11 (0.5, 58)	10 (0.1, 55)	2.6 (2.33, 2.87)^*f*^	0.4^*e*^
Morphologically normal sperm (%)	19	7.0 (1.0, 17.0)	6.8 (0.5, 17.0)	2.64 (2.39, 2.89)^*g*^	0.5^*h*^
Progressively motile sperm (%)	10	64 (28, 81)	61 (28, 80)	3,942 (3,524, 4,361)^*i*^	0.4^*j*^
Abbreviations: cFT, calculated free testosterone; NA, not applicable. 5th and 95th are percentiles. ^***a***^*p*-Values represent the difference between genotype groups tested by multivariate linear regression. ^***b***^By Mann-Whitney test. ^***c***^Dependent variables were ln transformed. ^***d***^Adjusted for BMI, smoking (number of cigarettes per day), alcohol consumption (units consumed in the week before participation), time of blood sampling, and age; also adjusted for type of assay in the inhibin-B model. ^***e***^Adjusted for ejaculation abstinence time. ^***f***^Dependent variables were cubic root transformed. ^***g***^Dependent variable was square root transformed. ^***h***^Unadjusted. ^***i***^Dependent variable was squared. ^***j***^Adjusted for time from ejaculation to semen analysis.					

Other confounders were considered but not included in the final model because they were not significant predictors of outcomes in the final models with serum hormones or semen quality as outcomes: ethnicity, BMI squared, birth weight, *in utero* exposure to tobacco smoke, previous or current diseases (varicocele, cryptorchidism, or sexually transmitted diseases), recent fever, recent use of medication, and season. BMI, time of day of urine sample collection, and alcohol intake were considered but were not generally significant predictors in models with urinary phthalate metabolites as outcomes. A *p*-value < 0.05 was considered statistically significant in all statistical tests and models. Data analysis was performed using PASW Statistics, vol. 18 (IBM, Armonk, New York, USA).

## Results

*Stratification by* FLG *genotype*. A total of 65 (7.5%) men were either heterozygous (*n* = 63) or homozygous/compound heterozygous (*n* = 2) for the three *FLG* loss-of-function variants examined (Table 3). These 65 men thus constituted the *FLG*-null carrier group. The observed genotype prevalence of the 2282del4 and 2447X polymorphisms did not deviate significantly from the expected prevalence under the Hardy-Weinberg equilibrium assumption (*p* = 0.55 and *p* = 0.86, respectively), whereas R501X differed significantly (*p* = 0.03) based on a single homozygous carrier (the calculated expected number was 0.13). The two men with homozygous null genotypes (one homozygous for R501X and one compound heterozygous for R501X and 2282del4) did not report atopic disease or use of medication that could be ascribed to atopic diseases, and their urinary phthalate concentrations, reproductive hormones, and semen-quality parameters were unremarkable. There were no significant differences in reported previous urogenital diseases (cryptorchidism, hernia, sexually transmitted diseases, or varicocele) between the two genotype groups. The frequency of self-reported atopic diseases was largely comparable between the *FLG* genotype groups (WT vs. heterozygous or homozygous null genotype): any atopic disease (24% vs. 22%), allergy or hay fever (15% vs. 17%), asthma (12% vs. 11%), and eczema (1.4% vs. 1.5%). Moreover, the use of medication prior to participation did not differ between the genotype groups; however, only a few young men reported medication use within the past 3 months [28 men used any antibiotics, 27 used any asthma or allergy medication, 32 used any medication for skin conditions (mostly acne medication), 28 used any analgesics, and 16 used any other type of medication].

**Table 3:** Genotype distribution in 861 men from the general population [*n* (%)].

Genotype	Homozygous	Heterozygous	WT
R501X	1 (0.1)	21 (2.4)	839 (97.4)
2282del4	0	34 (3.9)	827 (96.1)
R2447X	0	10 (1.2)	851 (98.8)
*FLG*-null	2 (0.2)^*a*^	63 (7.3)	796 (92.5)
^***a***^One person was compound heterozygous for R501X and 2282del4.			

Age, alcohol use, and smoking were not significantly associated with genotype group (Table 2). BMI was slightly lower in the *FLG*-null group than in the WT group (median 21.9 versus 22.6 kg/m^2^; *p* = 0.04).

FLG *and reproductive hormones and semen quality*. We found no significant association between the *FLG* genotype and the levels of reproductive hormones or semen quality.

*Urinary phthalate metabolite concentrations and* FLG *genotype*. All metabolites were detectable in urine in > 95% of participants except MiNP and MOP (mono-*n-*octyl phthalate), which were measurable in 49% and 34% of men, respectively. Urinary concentrations of MOP were very low. MCPP is a metabolite of di-*n*-octyl phthalate (DnOP), but also a minor metabolite of other phthalate diesters, making it difficult to relate this metabolite to exposure to any specific phthalate. Therefore, data on MOP and MCPP were not analyzed further. Phthalate metabolite concentrations and sums of diester phthalates by *FLG* genotype group are shown in Table 4. Urinary concentrations of phthalate metabolites for the entire group of men have been published previously (Joensen et al. 2012). All phthalate metabolites and sums of phthalate isomers and metabolites [ΣDBP_(i+n)_, ΣDEHPm, and ΣDiNPm] were correlated to each other (for all, *p* < 0.001).

**Table 4:** Osmolality-adjusted urinary concentrations (ng/mL) of phthalate metabolites by *FLG* genotype, and estimated percentage difference between genotype groups, with corresponding *p*-values, from final multivariate regression models.

Phthalate group	Phthalate metabolite	Osmolality-adjusted median (5th, 95th percentiles)		Estimated percent difference^*a*^ (95% CI)	*p*-Value^*a*^
		*FLG*-null	*FLG* WT		
LMW	MEP	116 (18, 751)	83 (17, 2202)	0.4 (–39, 39)	0.9
	MiBP	77 (31, 201)	61 (22, 171)	21 (4, 39)	0.02
	MnBP	42 (14, 118)	29 (9, 89)	33 (16, 51)	0.0002
	ΣDBP_(i+n)_	156 (65, 383)	117 (42, 319)	26 (9, 42)	0.003
	MBzP	46 (14, 171)	37 (10, 157)	24 (0.2, 47)	0.05
HMW	MEHP	5 (1, 15)	4 (0.5, 17)	13 (–12, 37)	0.3
	MEHHP	29 (12, 79)	23 (8, 76)	16 (–5, 37)	0.1
	MEOHP	19 (7, 45)	15 (4, 55)	20 (–1, 41)	0.06
	MECPP	20 (7, 55)	16 (6, 53)	14 (–5, 33)	0.1
	MiNP	0.7 (0.1, 4)	0.6 (0.1, 5)	–3 (–36, 30)	0.8
	MHiNP	6 (1, 26)	4 (0.9, 22)	25 (–2, 52)	0.07
	MOiNP	3 (0.7, 13)	2 (0.4, 11)	26 (–4, 56)	0.09
	MCiOP	8 (3, 55)	8 (3, 38)	14 (–7, 35)	0.2
	ΣDEHPm	96 (37, 231)	80 (29, 271)	16 (–3, 35)	0.1
	ΣDiNPm	24 (7, 154)	21 (6, 95)	16 (–6, 38)	0.2
All shown metabolites were detectable in urine in > 95% of participants, except MiNP (measurable in 49% of men). Abbreviations: LMW, low-molecular-weight phthalates often found in personal care products; HMW, high-molecular-weight phthalates used as plasticizers, infrequently found in personal care products. ^***a***^Data are from multivariate linear regression models using ln-transformed phthalate metabolite concentrations, adjusting for age, smoking, and year of participation; adjusted means are provided in Figure 1.					

**Figure 1:**
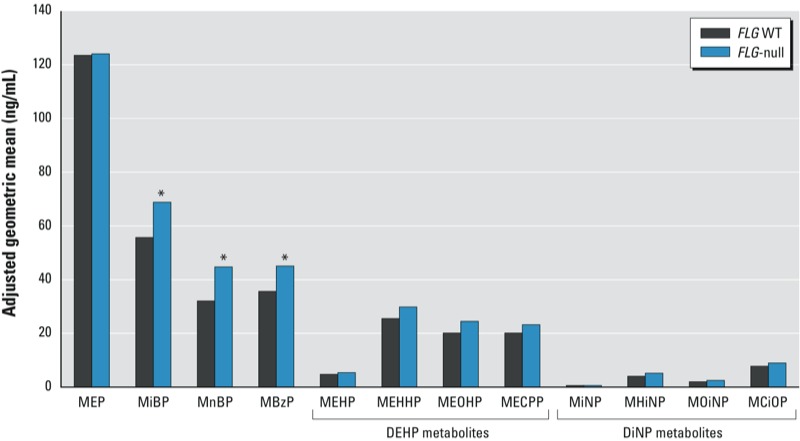
Adjusted geometric means of phthalate metabolites (predicted values for 19 years, nonsmoker, and participating year 2007) by *FLG* genotype. *FLG*‑null carriers (*n* = 65) had higher urinary excretion of common phthalate metabolites (from both LMW and HMW phthalates). *p < 0.05 compared with WT genotype.

*FLG*-null allele carriers had higher excretion of all phthalate metabolites—both LMW and HMW phthalates—after adjusting only for urinary dilution by osmolality (Table 4). After adjusting for other relevant confounders (age, smoking, and year of participation), this difference remained significant for the LMW phthalates MiBP (monoisobutyl phthalate), MnBP, and MBzP (monobenzyl phthalate) (Table 4, Figure 1). Differences between group means were substantial; for example. MnBP was 33% higher (95% CI: 16, 51%) in *FLG*-null allele carriers compared with WT carriers. For the HMW phthalates, *FLG*-null allele carriers had higher osmolality-adjusted levels for every measured metabolite and for ΣDEHPm and ΣDiNPm, but none of the associations were statistically significant after adjustment for year of participation, age, and number of cigarettes per day.

When the analyses were re-run including only men that were of ethnic European origin (participant and both of his parents born in a European country), estimates and *p*-values remained essentially unchanged (data not shown). Neither %MEHP nor %MiNP, which reflect phthalate metabolism, was associated with *FLG* genotype (data not shown).

## Discussion

Young healthy men with any of the three common *FLG*-null variants examined in the present study had significantly higher internal exposure to phthalate metabolites than men with WT *FLG* genotypes, as assessed by urinary phthalate metabolite concentrations. The higher phthalate levels in *FLG*-null carriers could be due to *a*) increased exposure to cosmetic products, *b*) enhanced penetration of chemicals across the skin barrier, or *c*) both. Use of skin moisturizers to treat xerosis may indeed be higher in *FLG*-null carriers (Sergeant et al. 2009). However, in the present study, *FLG*-null carriers had higher levels of metabolites of both LMW phthalates (commonly found in skin care products) and HMW phthalates (usually not present in personal care products). Urinary levels of LMW phthalates were higher than those of HMW phthalates, which is in accordance with studies in other populations (reviewed by Wittassek et al. 2011). The median urine concentration of MEP (monoethyl phthalate), which is the primary metabolite of the LMW phthalate DEP and the most frequently detected phthalate in personal care products, was considerably higher in the *FLG*-null variant group than in other men. However, after adjustment for urinary dilution, year of participation, age, and cigarette smoking, the association was not significant, in contrast with MnBP and MiBP, which are metabolites of LMW DBP isomers. In a study on topical application of DEP and DBP, Janjua et al. (2008) observed that *a*) urinary concentrations of the metabolite MEP were much higher after dermal application than were MnBP concentrations; *b*) MEP was cleared more quickly than MnBP; and *c*) compared with DBP, more of the applied amount of DEP was recovered in the urine (as MEP). This supports our hypothesis that enhanced permeability to DBP, and not only the increased exposure, may account for the difference in metabolite levels between the *FLG* genotype groups. Although we adjusted for confounding by several factors, the possibility of chance associations resulting from multiple testing or bias due to uncontrolled confounding cannot be ruled out. In addition, because we are not aware of similar studies, we cannot determine whether our findings would be consistent with other populations.

We found no significant differences in reproductive hormones or semen quality between *FLG*-null and WT carriers. We cannot exclude, however, that *FLG*-null carriers may also have a higher internal exposure to other factors, such as vitamin D, that may actually improve semen quality (Blomberg et al. 2011; Thyssen et al. 2012b).

We recently published associations between reproductive hormones and the proportions of DEHP and DiNP excreted as their respective primary metabolites (%MEHP and %MiNP, respectively) in this population (Joensen et al. 2012). In that study, several reproductive hormones were significantly associated with the proportions of %MEHP and %MiNP, but there was little evidence of significant associations of urinary phthalate metabolites or sums of phthalates with reproductive hormones or semen quality. In the present study, neither %MEHP nor %MiNP was associated with *FLG*-null genotype, suggesting that an individual’s pattern of metabolism of DEHP and DiNP was unrelated to *FLG*-null genotype.

Because phthalate diesters are rapidly metabolized and the metabolites are excreted mainly in the urine, with elimination half-lives of usually < 24 hr (Koch et al. 2005), measured phthalate levels reflect very recent exposure to the parent compounds (phthalate diesters). Measurement of metabolites in urine samples by the methods used in the present study is accepted as the state-of-the-art method for biomonitoring these substances. Despite the considerable day-to-day variation in excretion of phthalates, a single urine sample is reasonably predictive of exposure during a 3-month period (Hauser et al. 2004), which constitutes a reasonable exposure time to investigate effects on spermatogenesis and reproductive hormones. The *FLG*-null carrier frequency of 7.5% was consistent with previous Danish population-based studies (Thyssen et al. 2010). The questionnaires we used focused on reproductive disorders and not skin or atopic disorders; hence, we did not ask specifically for information about eczema or the use of medication for skin conditions that could be related to filaggrin deficiency. This may explain why the two homozygous individuals did not report such disorders, and why the prevalence of self-reported atopic dermatitis in the whole group was very low (1.4%). Although we did not find any significant associations between *FLG* genotype and reproductive hormone levels or semen-quality parameters, the number of *FLG*-null carriers in our population may have been too small to provide sufficient statistical power to detect small differences in these outcomes.

Our findings suggest that men with *FLG* loss-of-function alleles may be at risk of excessive chemical exposure due to differences in skin barrier function resulting from reduced *FLG* expression. The use of the phthalates DEHP, BBzP, and DnBP in the European Union is currently restricted but not banned. Although these phthalates are classified as reproductive toxicants, the use of DiNP is restricted only in certain toys and childcare items for children < 3 years of age (European Chemical Agency 2010). Immunotoxicological effects have been proposed for some phthalates, but it is currently unknown whether phthalate exposure may increase the risk of allergic disorders (Kimber and Dearman 2010). Although a recent cross-sectional study from Denmark showed that the prevalence of *FLG*-null variants increased from 12.9% in individuals with atopic dermatitis who were born in 1936–1949 to 19% among individuals with atopic dermatitis who were born in 1976–1988 (Thyssen et al. 2012a), one may speculate whether the increased phthalate exposure from skin care products over the second half of the 20th century may have played a role in the epidemic of atopic dermatitis. In fact, McFadden et al. (2009) proposed the “hapten-atopy” hypothesis, which suggests that an increased exposure to chemicals, particularly during pregnancy and the first year of life, may predispose to subsequent development of atopic disease. At present, clinical trials are being conducted to test the possibility of primary prevention of atopic disorders by using topical therapy with moisturizers (Irvine et al. 2011), but many such skin care products are not globally regulated by legislation and may contain phthalates (European Chemical Agency 2010). *FLG*-null carriers may constitute a susceptible group for which special attention to phthalates and other dermally applied chemicals in skin care products is warranted.

## Conclusions

We found that young healthy men with *FLG*-null variants had significantly higher internal exposure to phthalate metabolites than WT carriers, and results from this study raise important questions. Our findings must be replicated in other study populations, and additional work would be necessary to determine whether higher levels of urinary phthalates reflect greater external exposure (due to increased use of skin care products), greater internal exposure (due to increased absorption through the skin), or both. Moreover, it is unknown whether the possible increased skin permeability of *FLG*-null carriers applies to classes of chemicals other than phthalates. The question of a possible disruption of testicular function in *FLG*-null carriers cannot, with certainty, be answered by this study. In addition, the possibility of an increased uptake of dermally applied pharmaceuticals has never been investigated. This study highlights the importance of ensuring that excess exposure to chemicals that are potentially endocrine disrupting or otherwise harmful is not overlooked as a side-effect of the treatment aiming to restore a defective skin barrier.

## Supplemental Material

(123 KB) PDF
