# Supplementary material for: Associations of Filaggrin Gene Loss-of-Function Variants with Urinary Phthalate Metabolites and Testicular Function in Young Danish Men
Source: Environ Health Perspect. 2014 Jan 3;122(4):345–50. doi: 10.1289/ehp.1306720 (PMC3984221; doi:10.1289/ehp.1306720)
Supplement: (123 KB) PDF [file ehp.1306720.s001.pdf]

Environmental Health Perspectives doi:10.1289/ehp.1306720

Supplemental Material

Associations of Filaggrin Gene Loss-of-Function Variants with Urinary Phthalate Metabolites and Testicular Function in Young Danish Men

Ulla Nordström Joensen, Niels Jørgensen, Michael Meldgaard, Hanne Frederiksen, Anna-Maria Andersson, Torkil Menné, Jeanne Duus Johansen, Berit Christina Carlsen, Steen Stender, Pal Bela Szecsi, Niels Erik Skakkebæk, Ewa Rajpert-De Meyts, and Jacob P. Thyssen

Table of Contents

Supplemental Material, Table S1 page 2

Supplemental Material, Figure S1 page 3

Supplemental Material, Table S1. Genotype distribution by participation year, N (%), show that a higher frequency of *FLG* null carriers was observed in 2007 than in 2008 and 2009 (difference in frequency distribution between year is not statistically significant).

	Participation year	Homozygous	Heterozygous	Wild-type
R501X	2007	0	10 (4)	265 (96)
	2008	1 (0.3)	5 (2)	284 (98)
	2009	0	6 (2)	289 (98)
2282del4	2007	0	14 (5)	261 (95)
	2008	0	11 (4)	279 (96)
	2009	0	9 (3)	286 (97)
R2447X	2007	0	5 (2)	270 (98)
	2008	0	1 (0.3)	289 (100)
	2009	0	4 (1)	291 (99)
FLG null carriers	2007	0	29 (11)	246 (89)
	2008	1 (0.3)	17 (6)	272 (94)
	2009	1 (0.3)	17 (6)	277 (94)

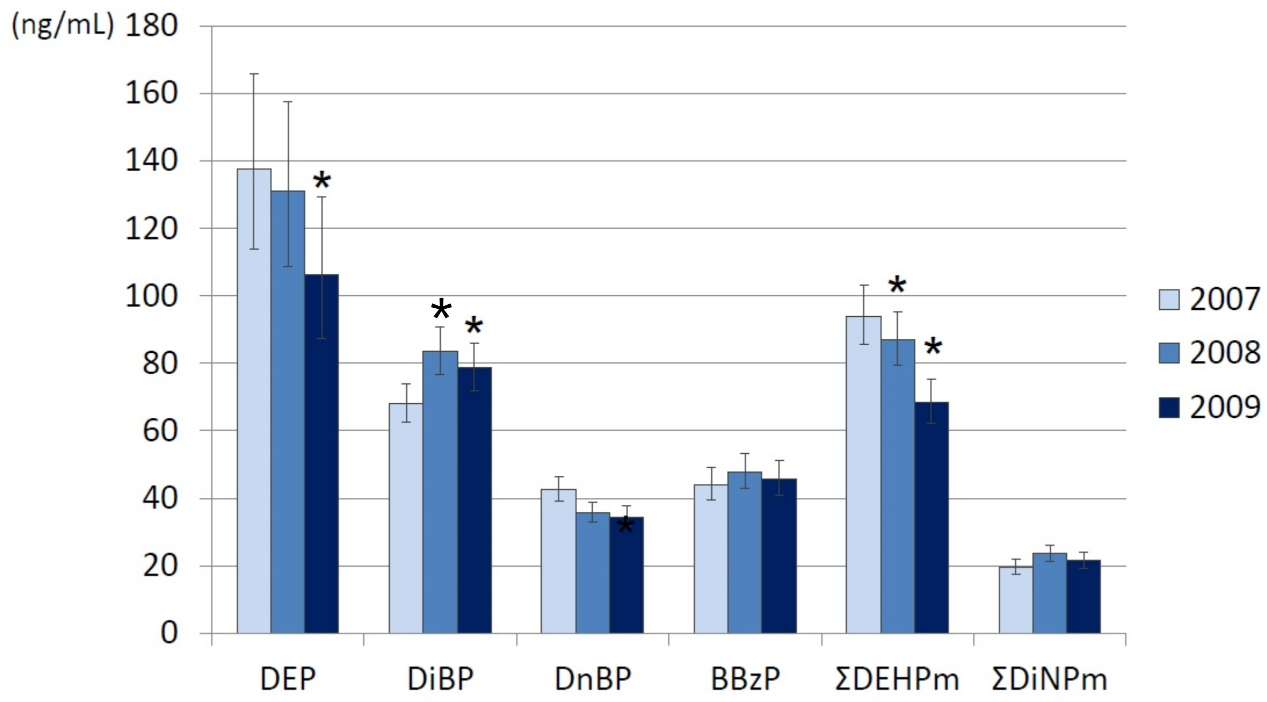

Supplemental Material, Figure S1. Phthalate excretion by year of examination in young Danish men (N = 861). Phthalate diesters (ng/mL) are calculated from urinary concentrations of their respective metabolites. Bars are geometric means (predicted values for a 19 year-old non-smoker). Whiskers are 95% CI for the geometric mean. *p < 0.05 for difference from 2007 levels.
